# Skin microbiome alters attractiveness to *Anopheles* mosquitoes

**DOI:** 10.1186/s12866-022-02502-4

**Published:** 2022-04-11

**Authors:** Alicia Showering, Julien Martinez, Ernest Diez Benavente, Salvador A. Gezan, Robert T. Jones, Catherine Oke, Scott Tytheridge, Elizabeth Pretorius, Darren Scott, Rachel L. Allen, Umberto D’Alessandro, Steve W. Lindsay, John A. L. Armour, John Pickett, James G. Logan

**Affiliations:** 1 Department of Disease Control, London School of Hygiene & Tropical Medicine, London, UK; 2 MRC-University of Glasgow Centre for Virus Research, University of Glasgow, 464 Bearsden Road, Glasgow, G61 1QH UK; 3 Department of Experimental Cardiology, University Medical Center Utrecht, Utrecht, the Netherlands; 4 VSN International Ltd, Hemel Hempstead, Hertfordshire, UK; 5 Department of Medical Statistics, London School of Hygiene & Tropical Medicine, London, UK; 6 Institute for Infection and Immunity, St George’s, University of London, London, UK; 7 Medical Research Council Unit The Gambia at the London School of Hygiene and Tropical Medicine, Fajara, Gambia; 8 Department of Biosciences, University of Durham, Durham, UK; 9 School of Life Sciences, University of Nottingham, Queen’s Medical Centre, Nottingham, UK; 10 School of Chemistry, Cardiff University, Cardiff, Wales, UK

**Keywords:** Malaria, Body odour, Skin microbiome, *Anopheles coluzzii*, Mosquitoes, Human attractiveness, Repellents, Diversity

## Abstract

**Background:**

Some people produce specific body odours that make them more attractive than others to mosquitoes, and consequently are at higher risk of contracting vector-borne diseases. The skin microbiome can break down carbohydrates, fatty acids and peptides on the skin into volatiles that mosquitoes can differentiate.

**Results:**

Here, we examined how skin microbiome composition of women differs in relation to level of attractiveness to *Anopheles coluzzii* mosquitoes, to identify volatiles in body odour and metabolic pathways associated with individuals that tend to be poorly-attractive to mosquitoes. We used behavioural assays to measure attractiveness of participants to *An. coluzzii* mosquitoes, 16S rRNA amplicon sequencing of the bacteria sampled from the skin and gas chromatography of volatiles in body odour. We found differences in skin microbiome composition between the poorly- and highly-attractive groups, particularly eight Amplicon Sequence Variants (ASVs) belonging to the Proteobacteria, Actinobacteria and Firmicutes phyla. *Staphylococcus 2* ASVs are four times as abundant in the highly-attractive compared to poorly-attractive group. Associations were found between these ASVs and volatiles known to be attractive to *Anopheles* mosquitoes. Propanoic pathways are enriched in the poorly-attractive participants compared to those found to be highly-attractive.

**Conclusions:**

Our findings suggest that variation in attractiveness of people to mosquitoes is related to the composition of the skin microbiota, knowledge that could improve odour-baited traps or other next generation vector control tools.

**Supplementary Information:**

The online version contains supplementary material available at 10.1186/s12866-022-02502-4.

## Background

Individual people who are more attractive to mosquitoes receive the most bites, resulting in higher risk of contracting often lethal vector-borne diseases, including malaria [24]. Human attractiveness to mosquitoes is partially determined by our skin microbiome, which contributes to differences in volatile organic compounds (VOCs) in body odour [45]. There are over 500 VOCs in skin secretions, including acids, alcohols, aldehydes, esters and ketones [8]. *Anopheles* mosquitoes, which transmit malaria, exhibit electrophysiological and behavioural responses to several of these VOCs, including heptanal, lactic acid, propanoic acid and 1-octen-3-one [4, 5, 7, 27, 40]. Synthetic odour blends containing human VOCs have been developed for use in mosquito traps, with some success [30].

Bacteria that naturally occur on the skin have been shown to contribute to body odour, producing microbial volatile organic compounds (mVOCs) that can affect attractiveness to mosquitoes [45]. Skin bacteria catabolise amino acids and lipids in body secretions into pungent short chain carboxylic acids [15]. Sweat incubated with bacteria has been shown to be more attractive to *Anopheles gambiae* than freshly-secreted sweat [3]. Specific mVOCs produced by bacteria on the skin have been identified and tested for attractiveness to mosquitoes [45] and volatiles have been associated with attractiveness to mosquitoes [42]. Our study goes further to link the skin microbiome, body odour and attractiveness to mosquitoes, to predict candidate metabolic pathways and therefore elucidate mechanisms that lead to differential attractiveness.

Butanoic acid, carbon dioxide, lactic acid and propanoic acid together elicit *Anopheles* attraction [42]. On the other hand, people less attractive to *Aedes aegypti* have been shown to produce more specific repellent volatile chemicals, including aldehydes (decanal, octanal, nonanal), and ketones (geranylacetone and 6-methyl-5-hepten-2-one) [22], and current evidence suggests these are natural repellents. Further understanding of attractive and repellent mVOCs could lead to the development of mosquito repellents with different modes of action, or be applied to improve current push-pull systems that incorporate compounds to repel mosquitoes away from hosts and attractants to lure them into traps [44].

Many factors contribute to variation in attractiveness to mosquitoes, including age, diet, sex, pregnancy, personal hygiene and parasite infection [21, 29, 33, 37, 40]. In some cases, these changes are associated with differences in skin microbiome diversity [47]. There is also evidence that attractiveness to mosquitoes is partly under genetic control [11], and it has been hypothesised that human genes may influence attractiveness through shaping the skin microbiome composition [48]. Since heterogeneities in mosquito biting between people can have significant epidemiological impacts [13, 43], it is important to understand the role of the skin microbiome in differential attractiveness.

Currently, the role the skin microbiome plays in human attractiveness to mosquitoes is not fully understood. Investigating differences in abundance of bacterial genera on the skin between people is critical in understanding if differences in attractiveness are due to differences in the skin microbiome. Applying chemical ecology techniques to identify mVOCs in body odour and correlating these with bacteria on the skin can be used to identify bacterial genera of interest. The aim of this study was to identify differentially abundant genera of bacteria between poorly- and highly-attractive groups, then predict metabolic pathways involved in body odour production and attractiveness to *Anopheles* mosquitoes.

## Results

### Study samples and population

In order to investigate the association between attractiveness to mosquitoes and skin microbiome composition, we collected skin swabs from the plantar aspect of participants’ feet and measured behavioural response of *An. coluzzii* mosquitoes to the odour of the participants. In order to minimise potential confounding factors such as gender or hormonal cycles [34], while ensuring sufficient numbers of participants to be included in the study, only post-menopausal females, aged between 50 and 90 years were recruited to control for known variation in attractiveness to mosquitoes.

### Global differences in the microbiome community between poorly-attractive and highly-attractive groups

From the behavioural data distribution we calculated relative attractiveness as the number of mosquitoes that chose the participant’s sock divided by the number that chose a trap. Highly-attractive (top quintile, 27 individuals) and poorly-attractive (bottom quintile, 28 individuals) groups were then selected for the analysis of skin microbiome composition. Co-variates were compared between the poorly- and highly-attractive groups prior to analysis (Supplementary Table 1). The median Faiths Phylogenetic Diversity (PD), a broad measure of alpha diversity or species richness within individuals, was on average lower in the highly-attractive group compared to the poorly-attractive group, although there was no significant difference in mean PD between the two groups (PD t-test *P* = 0.757) (Supplementary Fig. 1).

**Fig. 1:**
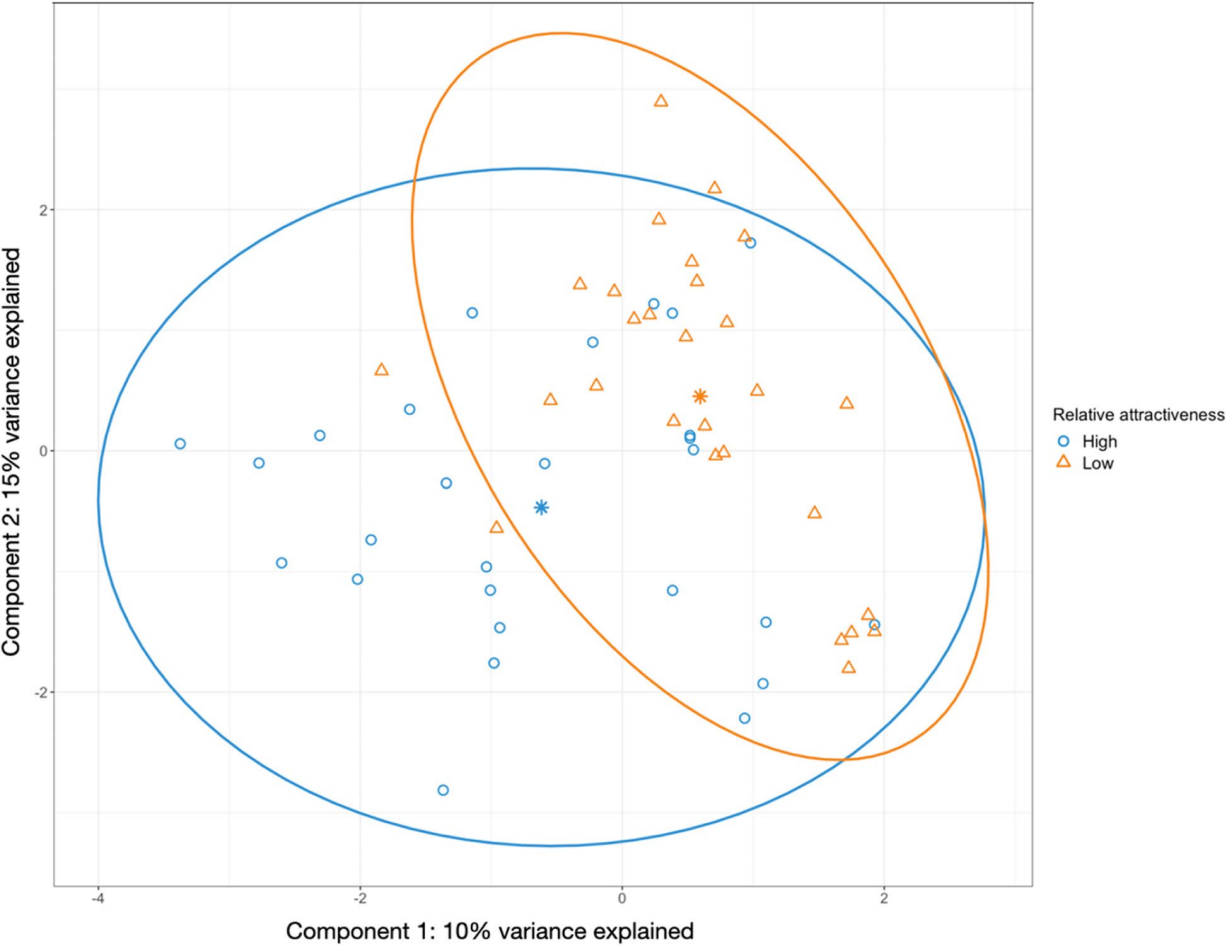
Differences in skin microbial composition (beta diversity) between the poorly- and highly-attractive groups. Each participant’s attractiveness to mosquitoes was measured using a bioassay, and differences in microbial composition between participants calculated using centralised log ratio transformed, DEICODE beta diversities from 16S rRNA data (see Methods). The sparse partial least squares discriminant analysis (sPLS-DA) sample plot shown was used to compare differences in microbial composition between the poorly-attractive group (orange) and highly-attractive group (blue). Individuals are presented by small triangles (poorly-attractive) or small circles (highly-attractive). Data were scaled (centred and standardised). The centroids (stars) represent the mean microbial composition on the first and second components for each group. The ellipse plots (large circles) represent 95% confidence intervals for the relative attractiveness groups

Sparse partial least squares discriminant analysis (sPLS-DA) was used to compare poorly- and highly-attractive groups based on beta diversity, a measure of variation in species diversity between individuals (Fig. 1). Components 1 and 2 combined explain 25% of the differences between attractiveness groups. The centroids for the groups are separated on the 1st and 2nd component, albeit with overlapping 95% confidence intervals. The separation between the groups was of borderline statistical significance (PERMANOVA, *P* = 0.055), suggesting weak evidence for a difference in microbial composition between groups.

### Differences in individual bacterial taxa between poorly-attractive and highly-attractive groups

**Fig. 2:**
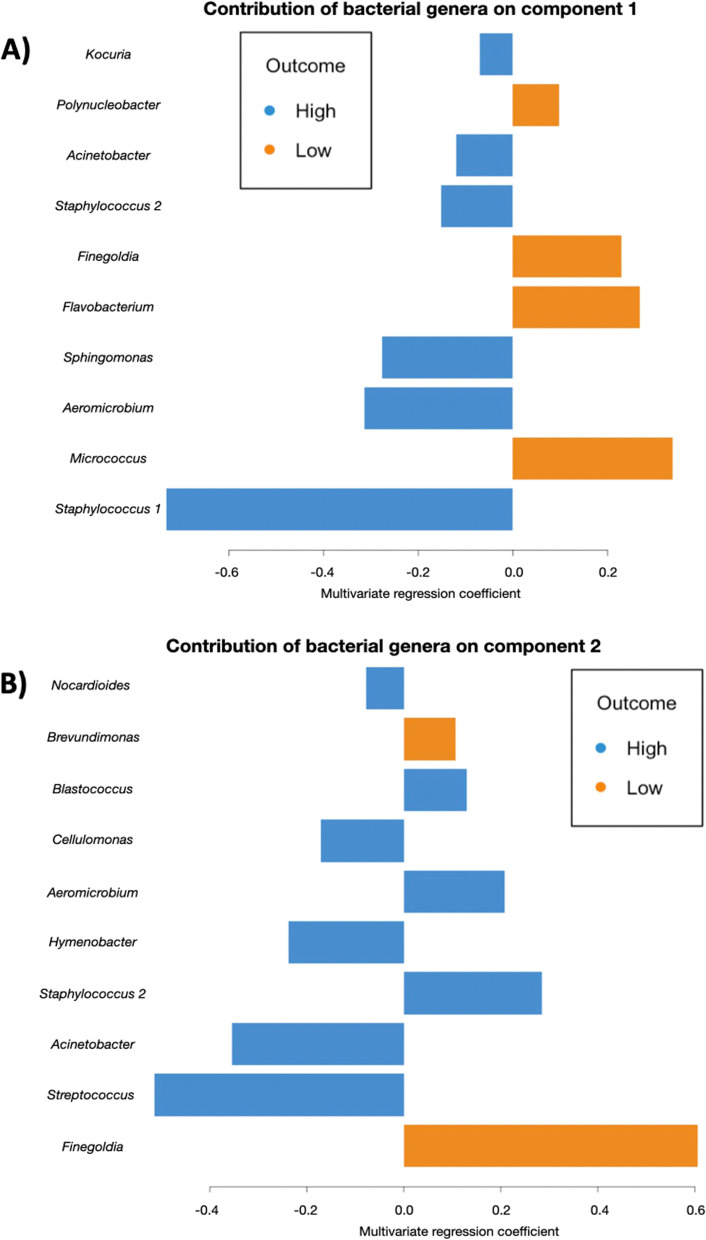
Bacterial genera with the greatest contribution to differences in microbiome composition between poorly- and highly attractiveness groups. The loading plot represents the 10 bacterial genera contributing the most to differences between attractiveness group on **A** component 1 and **B** component 2 of the sPLS-DA. Bars represent the loading weights or correlation coefficients of each bacterial genus to the components of the sPLS-DA. The direction of the bars (left or right) relates to the direction of the loadings in Fig. 1. Orange and blue bars indicate a higher abundance in the poorly- or highly-attractive group respectively. There are two Staphylococcus genera belonging to different families: Staphylococcus 1 belongs to the Planococcaceae family and Staphylococcus 2 belongs to the Staphylococcaceae family

The contribution of individual genera of bacteria to differences in microbiome composition between the attractiveness groups was investigated by selecting and ranking taxa by order of importance on the two components of the sPLS-DA model (Fig. 1). The strongest correlations (*r* > 0.5 or *r* < − 0.5) between relative abundance and global differences in microbiome between the attractiveness groups were observed for *Staphylococcus 1* (on component 1), *Finegoldia* (on component 2) and *Streptococcus* (on component 2) (Fig. 2). *Staphylococcus 1* and *Streptococcus* were more abundant among highly attractive participants, while *Finegoldia* was more abundant in the poorly-attractive group (Fig. 2). Among the top ten bacterial genera contributing the most to differences in microbiome composition, additional taxa showed weaker contribution such as *Acinetobacter* and *Micrococcus,* which were found to be more abundant in the highly and poorly attractive group, respectively.

**Fig. 3:**
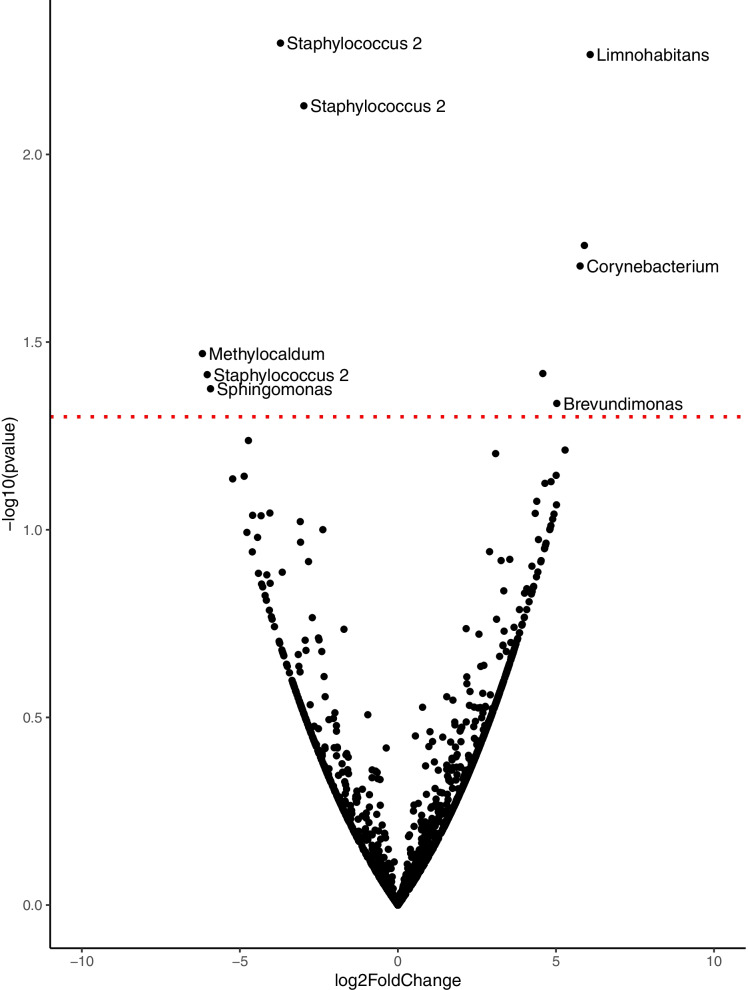
Differentially abundant bacteria between poorly- and highly-attractive groups. Volcano plot of amplicon sequence variants (ASVs, black dots). DESEQ2 was used to calculate log 2 fold changes i.e. if bacterial ASVs are more or less abundant in the poorly-attractive compared to highly-attractive group. X axis represents log 2 fold change abundance in the poorly-attractive compared to highly-attractive groups, with the biggest changes furthest from the centre. Y axis indicates the negative log-10 transform of the nominal *p*-value, i.e. increasing significance away from the origin. Red line represents *P* = 0.05 for exploratory purposes. ASVs above the red line are nominally significance and considered differentially abundant. Where genus level taxonomy is available and the ASV is above the red line it is labelled with genus level taxonomy

Differential abundance of bacterial genera between attractiveness groups was further tested using DESEQ2 (Fig. 3). We found 10 Amplicon Sequence Variants (ASVs) with abundances significantly different between the poorly- and highly-attractive groups, eight of which could be assigned taxonomy at the genus level: *Methylocaldum, Sphingomonas, Staphylococcus 2* (Staphylococcaceae family)*, Brevundimonas, Corynebacterium* and *Limnohabitans*. Among these, three ASVs belonged to the *Staphylococcus 2* genus; ASVs of *Staphylococcus 2* genus are increased four times or more in the highly- compared to the poorly-attractive group, suggesting that the presence of *Staphylococcus* is strongly associated with attraction to *Anopheles* mosquitoes. Three of the six genera were identified as differentially abundant between the attractiveness groups: *Sphingomonas, Staphylococcus 2* and *Brevundimonas* were also identified in the exploratory multivariate analysis in Fig. 2.

### Microbial associated VOCs involved in attractiveness to mosquitoes

**Fig. 4:**
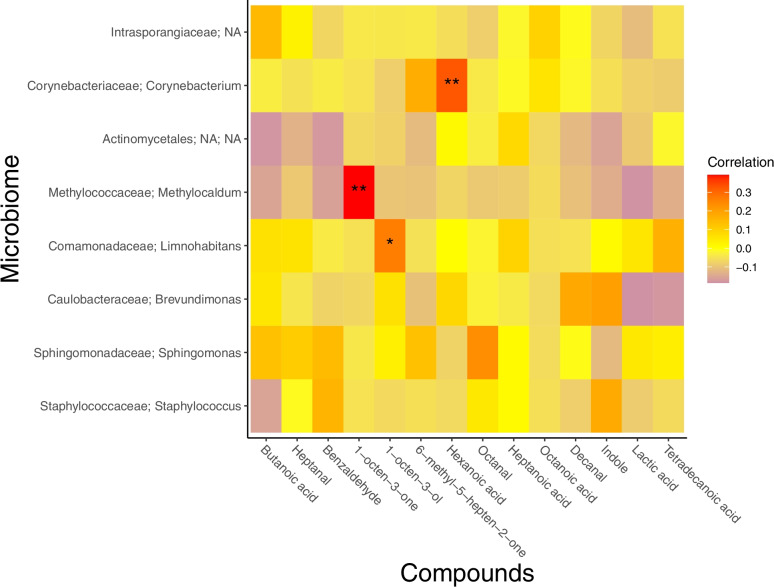
Heatmap correlation compounds from the literature and differentially abundant ASVs. Heatmap showing Pearson correlations between known compounds from the literature tentatively identified in our dataset and the differentially abundant ASVs identified, taxonomy assigned at family; genus level. Asterisks represent *p*-values from Pearson correlation, < 0.001 is represented as ** and < 0.05 as *

The levels of the differentially abundant bacterial ASVs were explored for associations with known compounds for which *Anopheles* mosquitoes have previously been shown to have olfactory receptors. *Anopheles* electroantennography (EAG) active, or behaviourally active compounds were extracted from the literature and included if considered to be microbial volatile organic compounds (mVOCs), according to mVOC 2.0 database [20] and VOCs were identified by GC (see methods). To investigate whether the known compounds were derived from people or the skin bacteria, we used a heatmap to display Pearson’s correlations between the ten differentially abundant ASVs and the known *Anopheles* active compounds [4, 5, 27, 40] (Fig. 4). The abundances of *Corynebacterium*, *Methylocaldum* and *Limnohabitans* showed significant positive correlations with the amount of hexanoic acid, 1-octen-3-one and 1-octen-3-ol respectively (Fig. 4). This suggests that the association between skin microbiome composition and attractiveness to mosquitoes could be partly mediated by the production of these three mVOCs by these bacterial ASVs.

### Elucidating metabolic pathways linked to attractiveness

**Fig. 5:**
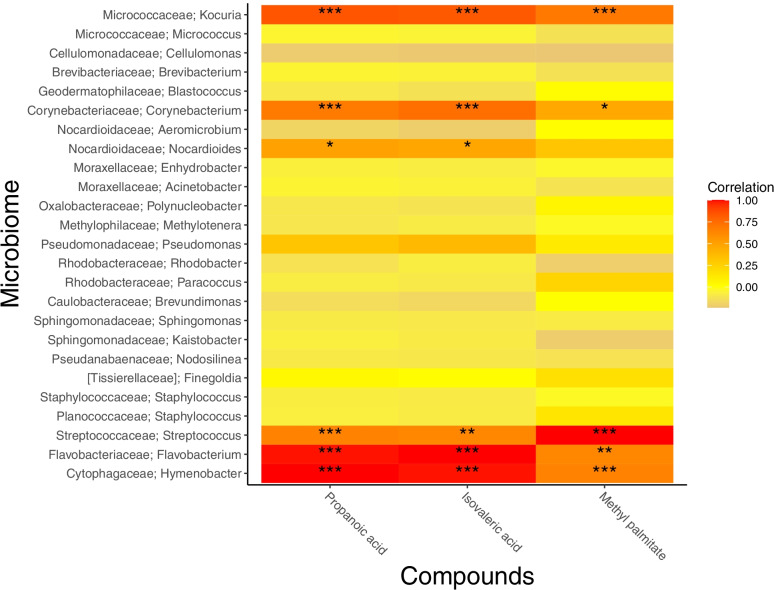
Heatmap compounds of interest identified and bacteria. Heatmap showing Pearson correlations between compounds of interest identified from the pathways and the 25 genera of bacteria identified in our samples that could be classified to the genus level, taxonomy at family; genus level. Asterisks represent *p*-values from Pearson correlation, < 0.0001 is represented as ***, < 0.001 is represented as ** and < 0.05 as *

We then predicted metabolic functions based on our 16S amplicon data. We compared pathway occurrences between the poorly- and highly-attractive groups to identify metabolic functions that were enriched. Metabolic pathways identified could be impacting attractiveness to mosquitoes. Supplementary Table 2 shows a subset of the differences in functional enrichment with log2 fold change > 1.5 or < − 1.5 i.e. 2.8 times increase or decrease in average pathway occurrence. Despite large log fold changes, there were no statistically significant differences in mean occurrence between the poorly- and highly-attractive groups. Although not significant, propanoate pathways were more common in the poorly-attractive group than the highly-attractive group. Indeed, the MetaCyc pathways PWY-5088 (L-glutamate pathway) and VALDEG-PWY (L-valinine pathway) were enriched in the poorly-attractive group compared to highly-attractive group, suggesting they could be involved in lower attractiveness to *Anopheles* mosquitoes. In the L-glutamate pathway, pyruvic acid is a repellent candidate that can produce propanoic acid. Previous studies have shown *Anopheles* have olfactory receptors that respond to propanoic acid [4]. Propanoic acid is an mVOC, which we found to be strongly associated with *Hymenobacter* bacteria in our study (*P* < 0.0001) (Fig. 5). From the degradation of L-valine, there are many potentially repellent compounds, including 3-methyl-2-oxobutanoic acid, isobutanoic acid, methylacrylic acid, (*S*)-hydroxyisobutanoic acid and propanoic acid, which warrant further analysis. In our volatile samples we found that propanoic acid, isovaleric acid and methyl palmitate were positively associated with *Hymenobacter*, *Flavobacterium*, *Kocuria*, *Corynebacterium* and *Streptococcus*, as indicated by red squares in Fig. 5.

## Discussion

In this study we explored the difference in skin microbiome composition between groups of participants that were poorly- and highly-attractive to *Anopheles* mosquitoes. The data here support previous research that showed there are differences in skin microbiome composition between attractiveness groups, and we have identified specific bacterial ASVs that are differentially abundant between these groups. Further, we have putatively identified metabolic pathways that are enriched in poorly-attractive participants, and the functional potential of compounds in these pathways as mosquito repellents.

We identified 10 differentially abundant bacterial ASVs between the poorly- and highly-attractive groups, and were able to assign genus level taxonomy to eight of these. In particular, three *Staphylococcus 2* ASVs were found to be more abundant in group of individuals highly-attractive to *An. coluzzii* than those who were poorly-attractive. This result supports our multivariate analysis presented here and the findings published by Verhulst et al. [46, 47], which found *Staphylococcus* spp. were more abundant in the highly-attractive group compared to poorly-attractive [47]. This suggests that *Staphylococci* within the Staphylococcaceae family (*Staphylococcus* genus) produce mVOCs that are detectable and attractive to *Anopheles*.

In addition, we found an ASV belonging to the genus *Corynebacterium* as differentially abundant, with a higher abundance in the poorly-attractive group than the highly-attractive group. This indicates that *Corynebacterium* may produce compounds that are not attractive or are repellent to *An. coluzzii*. Furthermore, we found higher *Corynebacterium* abundance was associated with higher levels of hexanoic acid in body odour; hexanoic acid has previously been suggested as a repellent at low doses [42]. This finding contrasts with those of Verhulst et al. [48], who tested bacterial species individually and reported that *Corynebacterium* produce attractive volatiles [45]. Bacteria may produce attractive VOCs when cultured individually, whereas in a blend they may interact antagonistically and have no effect on attractiveness. Alternatively, there may be differences in VOCs produced by different species of *Corynebacterium* or when these are grown in culture media. Our findings suggest *Corynebacterium* abundance is important in differentiating poorly- and highly-attractive participants, and is associated with production of hexanoic acid, which may be a contextual repellent.

We identified four other differentially abundant ASVs of the Proteobacteria phylum that have not been previously reported in human attractiveness to *Anopheles* that could be assigned to specific genera: these were *Methylocaldum, Sphingomonas, Brevundimonas* and *Limnohabitans*, which warrant further investigation. *Sphingomonas* and *Brevundimonas* have previously been identified as attractive in oviposition sites to *Aedes* [35, 36]. We found associations of these bacteria with the known *Anopheles* active VOCs these bacteria may produce (1-octen-3-one, octanal and 1-octen-3-ol), which could improve current blends being used in vector control tools to mimic human body odour [46].

We identified two propanoate metabolic pathways, L-glutamate and L-valine pathways, predicted to be involved in attractiveness from our 16S data. Both pathways were enriched in the poorly-attractive group compared to the attractive one, suggesting they may produce repellents. The L-glutamate pathway produces pyruvic acid which can produce propanoic acid, a known *Anopheles* attractant [42]. We found propanoic acid associated with *Hymenobacter, Flavobacterium*, *Kocuria*, *Corynebacterium* and *Streptococcus*. We also identified several repellent candidate VOCs in the L-valine pathway that warrant further investigation. It would be interesting to apply shotgun metagenomics approaches to the skin microbiome for precise classification of bacteria to species level and identify function encoded in bacterial genomes, to further understand the metabolic potential of the skin microbiome and determine if the propanoate pathways identified here have a role in attractiveness. The mVOCs produced through these metabolic pathways could be making these participants naturally repellent, reducing the number of mosquito bites they receive and, therefore, risk of contracting vector-borne disease.

Additionally, we found alpha diversity was not significantly different between attractive groups, which contrasts to Verhulst and colleague’s study [46, 47] that showed significant differences in phylogenetic diversity. Species richness does not appear to be correlated with human attractiveness to *Anopheles* mosquitoes.

This study has a few limitations. First, the bacterial yield was relatively low as skin microbiome swabs are low biomass samples, which was likely to contribute to an increased number of contaminant reads. Nevertheless, we attempted to mitigate this issue by sequencing positive and negative controls and filtering out potential contaminants using the literature in addition to statistical tools, a step that is generally overlooked in metabarcoding studies. Second, the sample size for this study was larger than previous studies in this area but it is possible that we could still have limited power to detect some differences. Our thresholds for detecting differentially abundant ASVs were conservative for the exploratory analysis presented here, meaning there is a risk of some false positive results. Thirdly, the skin microbiome is expected to change over time and it is difficult to control for interpersonal variation due to environmental factors such as diet, hygiene and cosmetic use [12]. Further experimental and longitudinal work is needed to control for some of the potential confounding effects in this study and to functionally validate the candidate bacteria and putative mVOCs that we have tentatively identified. Another limitation of our analysis is that sample selection was restricted to post-menopausal female twins in the UK. Since there is some evidence that age and sex may be associated with attractiveness to mosquitoes, our results may only be relevant to this particular demographic.

Further understanding of the skin microbiome and VOCs produced could be used to focus vector control on those that are highly-attractive. Heterogeneities in biting between people have significant epidemiological impact, as those that are most attractive receive the most bites [13, 43]. Targeting those that are bitten the most relies on finding them. Trained detection dogs or devices could be used to find the individuals that drive most of the infection in communities, and protecting or treating these individuals could be more efficient than reaching 100% coverage with vector control tools [14]. Improved push-pull systems using bacterial and fungal VOCs could be used in the field to trap mosquitoes: previous push-pull systems using repellent blends in the eaves of homes and attractant traps outside have reduced mosquito house entry by more than 50% [28]. Alternatively, products containing repellent mVOCs, could be a durable treatment with potential to be used as mosquito repellents.

## Conclusions

In summary, the findings presented provide evidence that bacteria on the skin differentiate human attractiveness to mosquitoes. We have identified differentially abundant bacteria, and several new bacterial genera identified here warrant further investigation. We have predicted metabolic pathways that may be involved in attractiveness and hypothesised the role of compounds in these pathways on human attractiveness to mosquitoes. Future studies using shotgun metagenomic analysis of the microbiome are needed to further elucidate the mechanisms responsible for production of odour that is attractive to mosquitoes.

## Materials and methods

### Recruitment

The study was approved by the London School of Hygiene & Tropical Medicine Research Ethics Committee (approval number 14500). Written informed consent was obtained from all 176 human volunteers (88 twin pairs) selected from the TwinsUK database (see Methods). Thirty-eight monozygotic (MZ) and 50 dizygotic (DZ), white European, post-menopausal, female twin pairs between 50 and 90 years were recruited from the TwinsUK database from the Department of Twin Research, Kings College London (ethics reference E892). Twins were chosen because we were also interested in investigating the impacts of genetic backgrounds, the analysis of which will be published separately. Volunteers were required to fill in a questionnaire before visiting for sample collection to collect metadata on covariates: twin type, sex, height, weight, ethnicity, diet, lifestyle and medication. Participants were asked to avoid alcohol, strong-smelling food and wearing skin-care products on their feet for 48 h prior to sample collection. They were provided with odour-free soap (Simple, Unilever) to wash. Descriptive analysis was used to compare the characteristics of the women between monozygotic and dizygotic twin pairs prior to analysis (Supplementary Supplementary Table 1).

### Attractiveness of body odour to mosquitoes by dual-choice olfaction

#### Body odour collection

Body odours were collected for 7–8 h overnight on prewashed (using 70% ethanol) nylon socks (100% polyamide, 15 deniers, Marks & Spencer). Cotton gloves were worn while handling the socks. Participants were asked to wash their feet with the odour-free soap provided before putting on the socks. Socks were removed by participants and stored at − 20 °C in sterile glass vials until use in an olfactometer bioassay.

The foot was chosen as the sampling site as *Anopheles* mosquitoes have been shown to have a preference for feeding on the feet compared to other body parts [9, 49]. Feet have been used elsewhere for investigation of semiochemical signatures associated with attraction to mosquitoes [32, 40]. *Anopheles* preference for the feet correlates with eccrine sweat-gland densities, which are known to be associated with the skin microbiome composition [19, 48].

#### Mosquitoes

All bioassays used colony-reared non-blood fed, female *Anopheles coluzzii* mosquitoes (N’Gousso strain) aged 5–8 days old. Mosquitoes were collected before the experiment and given 1 h to acclimatise. Prior to the experiment, mosquitoes were maintained at 26 ± 1 °C and 70% humidity under a 12:12 light dark cycle.

#### Behavioural assay for attractiveness of odour samples

**Fig. 6:**
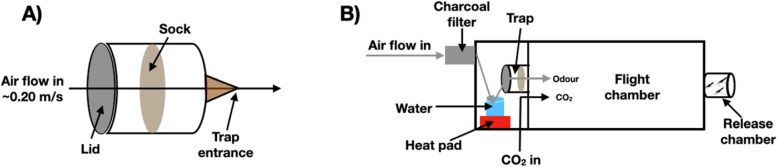
Diagram of the dual choice olfactometer. **A** The trap in which the nylon sock was placed. **B** The dual choice olfactometer from the side. Socks were placed on a wire frame inside the trap, and air flowed through the sock, carrying the body odour into the tunnel

Two identical dual-port olfactometers (Fig. 6B; Tupola, Wageningen University) were used to determine the attractiveness of odour samples from the twins. The olfactometers consisted of a large flight chamber (160 × 60 × 43 cm) [18]. Charcoal-filtered, pressurised air was heated, humidified and pumped into the flight chamber at a rate of 0.20 ± 0.01 m/s. To activate mosquito host-seeking behaviour, the air stream was supplemented with 5% CO_2_, released at the entrance, below each trap at a rate of 175 ml/min to mimic the levels in human breath [7]. In each trial, one nylon sock worn by a participant was placed in a trap and an unworn control sock was placed in the other (Fig. 6A). Mosquitoes were released from the release chamber at the opposite end of the flight chamber to the traps (Fig. 6B). Twenty mosquitoes were released per bioassay and were given 20 min to make a choice between the two traps. Experiments were performed during the last 4 h of dark phase, in a dimly lit room to mimic moonlight (temperature 27 ± 1 °C). After 20 min mosquito choice was recorded. Experiments were repeated twice for each sample, once per olfactometer. The sequence of samples was randomised for each replicate and sock samples were randomly assigned to the right or left port.

#### Data cleaning

All twin data was filtered to have a relative response more than 35%, i.e. at least 35% of mosquitoes needed to make a choice of entering one of the traps for the replicate to be included in the analyses. Relative attractiveness was calculated as the proportion of mosquitoes that selected the odour sample over the total number of mosquitoes collected in both trapping devices. It was adjusted for the replicate, temperature, humidity and time of day of the test. Relative attractive categories were coded based on top quintile being highly-attractive and bottom quintile poorly-attractive where not otherwise stated.

### Volatile odour profile analysis

#### Collection of volatile odour samples by air entrainment

Participants were asked to avoid washing their feet and lower legs on the day of sample collection. For each participant the right foot was placed in a prepared bag (Roasting Bags, 25 × 38 cm; Toastabags) and clipped shut around the calf. Bags were fitted with Swagelok fittings at opposite corners of the bag, and connected to polytetrafluoroethylene (PTFE) tubing. Charcoal filtered air was pumped into the top of the bag at 700 ml/min and vacuumed out the bottom at 600 ml/min [7]. The system was purged for 15 min before fitting a porapak filter (Porapak Q, mesh 50/80; Supelco Analytical) and air entraining for 120 min. At the end of sample collection, the porapak filter was removed and temporarily stored in clean stoppered vial before being sealed in a clean glass ampoule under charcoal-filtered nitrogen.

#### Analysis of odour profiles by gas chromatography

The collected volatiles were eluted from the porapak filters with 800 μl redistilled diethyl ether and concentrated to ~ 50 μl under a stream of charcoal filtered nitrogen [7]. A 4 μl sample was injected onto a 50 m non-polar polydimethylsiloxane (HP1) column (50 m × 0.32 mm, solid phase thickness 0.52 μm) in a gas chromatogram (Agilent Technologies 7890A Instrument fitted with a cool-on-column injector, nitrogen carrier gas and flame ionisation detector). The GC cycle consisted of: 40 °C for 30 s, raised by 5 °C per min to 150 °C, held for 1 min, raised by 10 °C per min to 230 °C, held for 40 min. Traces were integrated using ChemStation software (Agilent Technologies). Retention times were used to calculate Kovats retention index (KI) relative to a standard series of n-alkanes (C7-C25) using the equation: KI = 100((log_10_RtX–log_10_ Rtn)/(log_10_Rtn + 1–log_10_ Rtn)) +100n. Where RtX is the retention time for the compound of interest, Rtn is the retention time for the alkane before the compound of interest, and Rtn + 1 is the retention time for the alkane after the compound of interest. Peaks were manually aligned across samples. The average peak area of the alkane standards (C8-C25) was used to calculate the amount (in nanograms) of each analyte and the total amount of analytes in each injection, and this was multiplied by the total extract amount to calculate actual amounts per sample.

#### Data cleaning

Volatile peaks were filtered to remove low abundance VOCs and any VOC present less than 10 times across all samples was removed to reduce sparsity. Twin pair 71,721 and 71,272 were removed as there was no data in the sample for twin 71,721, most likely due to an error from running the sample on the GC.

#### *Anopheles* active compounds

The list of known EAG or behaviourally active *Anopheles* compounds was obtained from the literature [4, 5, 27]. We checked if compounds were known bacterial mVOCs, using mVOC 2.0 database [20]. We ran compounds that were commercially available on our GC to get the KI value which allowed us to identify the known compounds in the samples. All compounds ran are presented in Fig. 4: butanoic acid, heptanal, benzaldehyde, 1-octen-3-one, 1-octen-3-ol, 6-methyl-5-hepten-2-one, hexanoic acid, octanal, heptanoic acid, octanoic acid, decanal, indole, lactic acid and tetradecanoic acid. Tentative KIs were confirmed by co-injecting known compounds with samples and looking for an increase in peak area.

### Identification of skin bacteria

#### Collection of skin microbiome samples

Two swabs were collected from the sole of each of the 176 participant’s left foot to sample the skin microbiome on the same day as the volatiles and socks were collected. Sterile PTFE rings (20 mm diameter) were placed on the foot to ensure the same amount of surface was sampled for each participant. A sterile buccal swab (Isohelix) was pre-moistened in specimen collection buffer (Tris buffer [pH 7.9], 1 mM Ethylenediaminetetraacetic acid, 0.5% Tween-20) and rubbed on the foot surface in circular motions for 30 s. Swabs were placed into specimen collection buffer and stored at − 80 °C until DNA extraction.

#### DNA extractions and 16S amplicon sequencing

DNA was extracted using the DNeasy® PowerSoil® Pro Kit (QIAGEN, Hilden, Germany) according to manufacturer’s instructions with some modification. The modifications were to add additional cell lysis steps at the beginning and to elute in 60 μl of solution C6 to improve the DNA yield. DNA concentrations were measured using a Qubit® 2.0 Fluorometer. PCR amplification of the V3/4 region of the 16S rRNA gene using universal primers, 314F and 805R [17] was carried out by Polo GGB. Twenty five microliter PCR reactions contained 2.5 μl microbial DNA (< 5 ng/μl), 5 μl of forward and reverse primer (1 μM) and 12.5 μl KAPAHiFi HotStart ReadyMix (X2). Four positive controls (DNA and microbial community standards from ZymoBIOMICS) and two negative controls (swabs only) were sequenced alongside the 176 samples to control for cross-contamination in the downstream bioinformatic analyses. Amplification was performed using a PCR consisting of an initial denaturation (95 °C for 3 min), followed by 25 cycles of: Denaturation (95 °C for 30 s), annealing (55 °C for 30 s) and elongation (72 °C for 30 s) followed by a final elongation (72 °C for 5 min). PCR products were purified to remove free primer and primer dimers using AMPure XP beads and used as templates in a second PCR to attach dual indices and Illumina sequencing adapters, using Nextera XT Index Kit. The AMPure XP beads were used to clean up the final library before validation and quantification. The resulting libraries were validated using a Fragment Analyzer (High Sensitivity Small Fragment Analysis Kit) to check size distribution. The concentration of library samples was defined on the basis of the Qubit® 3.0 Fluorometer quantification and average library size. Indexed DNA libraries were normalised to 4 nM and then pooled in equal volumes. The pool was loaded at a concentration of 4 pM onto an Illumina Flowcell standard with 12.5% of Phix control. The samples were then sequenced using the Illumina MiSeq V2, to generate 2 × 250 base pair paired end reads.

#### Bioinformatics

Amplicon libraries were analysed using QIIME 22019.4 [2]. The raw reads were demultiplexed and quality-filtered, then denoised using DADA2 (q2-demux). Singletons and known 16S contaminants (mitochondria, chloroplasts) were removed. The sequencing depth for retaining samples was limited to a minimum of 2500 sequencing reads per sample, 43 samples were discarded. ASVs were aligned with mafft via q2-alignment [16] and a phylogeny constructed with fasttree2 via q2-phylogeny [38]. In the statistical software R a phyloseq object was created [26] and the decontam package [6] used for prevalence-based contaminant filtering. Based on the literature, additional genera likely to be contaminants were identified and removed if not previously reported on human skin. Alpha diversity metrics (bacterial diversity within samples: observed operational taxonomic units (OTUs), Faith’s Phylogenetic Diversity and Shannon’s diversity index), were estimated using q2-diversity after rarefying samples to 2500 sequences per sample. Beta diversities (bacterial diversity between samples) were later estimated using compositional methods, DEICODE QIIME2 plugin [25]. A bespoke classifier was created using the greengenes 13_8 database, naïve Bayes method on QIIME2 2019.4 and used to assign taxonomy to the ASVs using q2-feature-classifier [1] by aligning to the greengenes database, trimmed to the V3/V4 region.

#### q2-picrust2

We used q2-picrust2 [10] on our ASV feature table and sequences file to predict metabolic pathways from our 16S amplicon data. We set the nearest-sequence taxon index cut off to 2, to eliminate possibly problematic sequences. We used the mp hidden-state prediction method as recommended by the authors to predict MetaCyc pathway abundances. We then calculated log2 fold change between the attractiveness groups. We subset pathways which had a log2 fold change > 1.5 or < − 1.5 and looked at the pathway details of these MetaCyc pathways using https://metacyc.org which allowed us to identify pathways that could be of interest, those present in bacteria in our samples and producing VOCs. We checked the compounds produced were MVOCs using MVOC2.0 (http://bioinformatics.charite.de/mvoc/index.php).

#### Data cleaning

The filtered ASV table, taxonomy, rooted tree and metadata were imported to the statistical software R (version 3.6.3) [39] and a filtered phyloseq object was created. ASVs present in less than 10% of samples were filtered out using prune_taxa, which reduced the number of features to 419. Taxa bar plots of relative abundance data were generated at phyla and genera levels to look at the diversity. A centralised log ratio (clr) transformation was applied (took log of the ratio between observed abundance and geometric mean) and the data was agglomerated at the genus level. DEICODE QIIME2 plugin [25] was used to calculate beta distances, a form of Aitchison distance robust for high levels of sparsity (many zero values). DEICODE uses robust centred log ratio transformation (log transforms the nonzero values and then centres the data) and the transformed table is used as the input for matrix completion. The output was visualised as a sPLS-DA to identify genera of bacteria explaining differences between attractiveness groups, highly-attractive group (*N* = 27) and poorly-attractive group (*N* = 28). Cross validation predicted tuning to top ten genera on the first two components.

### Statistical analysis

#### General alpha beta analysis

We analysed alpha and beta diversities for associations with attractiveness. Spearman correlation tests were used to test if highly-attractive people have a higher alpha diversity, corresponding to a diverse skin microbiome, or a lower alpha diversity, with a less diverse skin microbiome.

#### Sparse partial least squares – discriminant analysis (sPLS-DA)

Beta diversities were visualised using sparse partial least squares-discriminant analysis (sPLS-DA), a multivariate analysis tool to show differences in microbial composition between highly- and poorly-attractive groups [41]. The number of components and variables on each component were selected by cross-validation. The contributions of individual genera of bacteria and volatiles on differential attractiveness was investigated by looking at the loadings (correlations with the discriminant function of the sPLS-DA) on first and second components. PERMANOVA was then used to test for a difference in microbial composition between attractiveness groups [31].

#### Differential abundance

We used DESEQ2 [23] to find differentially abundant genera of bacteria between attractiveness groups. We used the significance cut off α = 0.05. This tool obtains maximum likelihood estimates for ASV log fold change between two groups using a negative binomial GLM, then apply Bayesian shrinkage to obtain shrunken log fold changes. Subsequent tests between the attractiveness groups to obtain significances are Wald tests.

## Supplementary Information

**Additional file 1.** Supplementary results.

## Data Availability

Raw data is available at Zendo: 10.5281/zenodo.6366622.

## Abbreviations

VOCs: Volatile organic compounds
MZ: Mono-zygotic mVOC: microbial volatile organic compound
DZ: Di-zygotic
OTU: Operational taxonomic unit
ASV: Amplicon sequence variant
mVOC: Microbial volatile organic compound
sPLS-DA: Sparse partial least squares discriminant analysis
PD: Phylogenetic Diversity
EAG: Electroantennography
