# Supplementary material for: Skin microbiome alters attractiveness to Anopheles mosquitoes
Source: BMC Microbiol. 2022 Apr 11;22:98. doi: 10.1186/s12866-022-02502-4 (PMC9004177; doi:10.1186/s12866-022-02502-4)
Supplement: Supplementary file 1 — Additional file 1. Supplementary results. [file 12866_2022_2502_MOESM1_ESM.docx]

**Supplementary results.**

1. **Descriptive analysis**

The characteristics of the participants were compared between the poorly- and highly-attractive groups of participants prior to further statistical analysis (Supplementary Table 1).

Supplementary Table 1: Descriptive analysis comparing age categories, zygosity, BMI, sex and smoking status between the poorly- and highly-attractive groups.


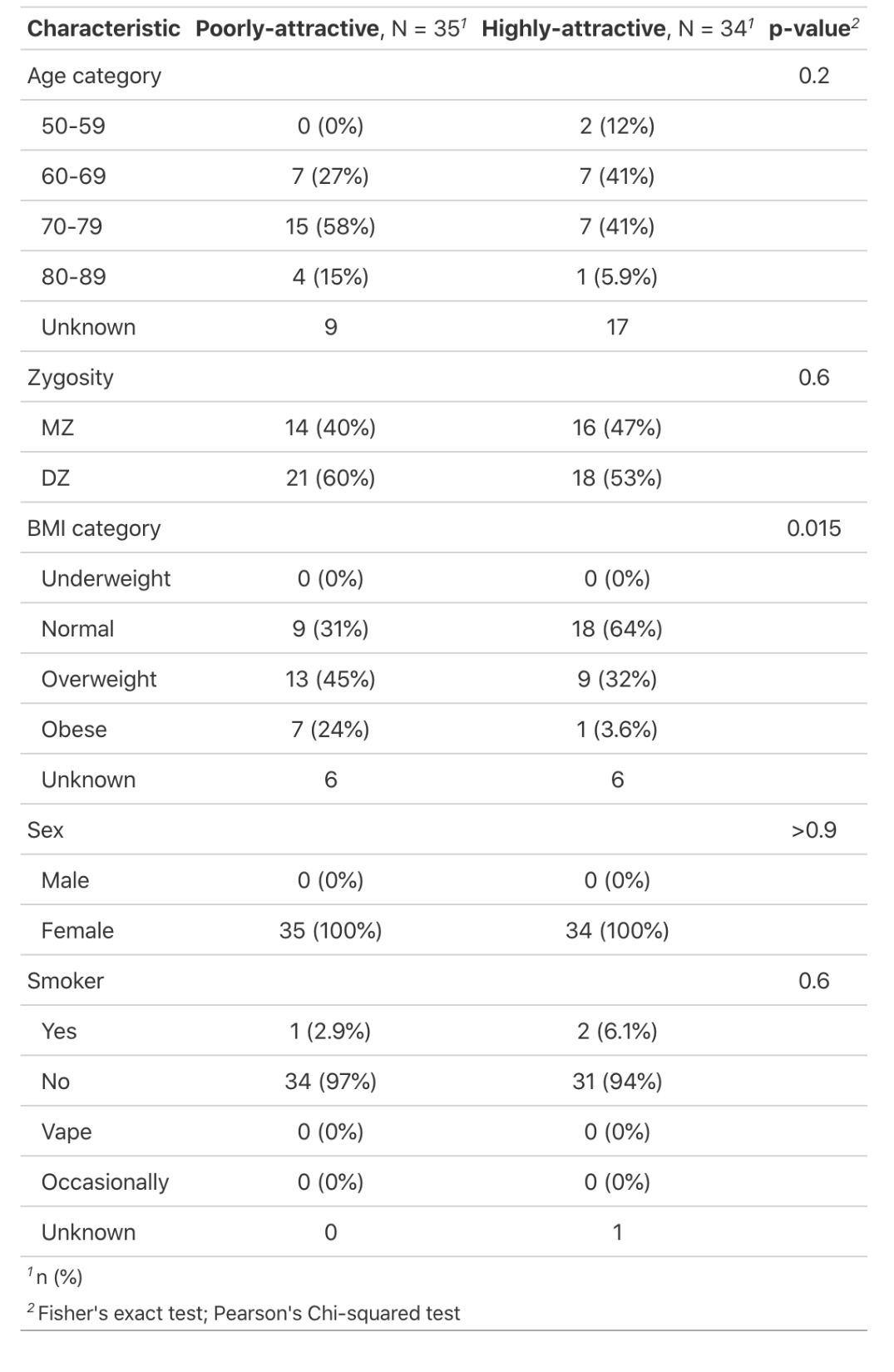


1. **Alpha diversity**

The median alpha diversity is lower in the highly-attractive compared to poorly-attractive group but there is no statistical evidence of a difference in means (P = 0.757).


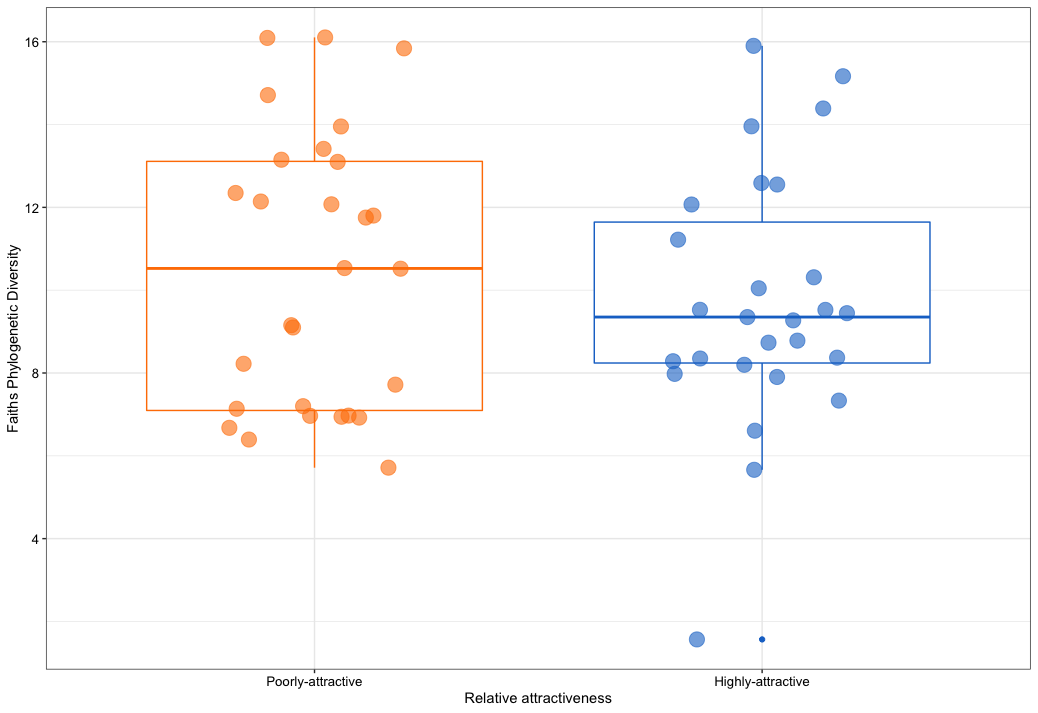


**Supplementary Figure 1: Differences in alpha diversity between the poorly- and highly-attractive groups.** Box plots of Faiths Phylogenetic Diversity (PD t-test P = 0.757).

1. **DESEQ2 table:**

Supplementary Table 2 presents detailed statistical results from DESEQ2 analysis to identify differentially abundant ASVs between the poorly- and highly-attractive groups.

Supplementary Table 2: DESEQ2 results for differentially abundant ASVs. 10 differentially abundant ASVs, eight of which could be assigned taxonomy to genus level. Base mean is the average normalised count value divided by the size factors over all samples. Log 2 fold change is the effect size, how much each ASV has changed in poorly-attractive compared to highly-attractive on log scale to base 2. lfcSE gives the uncertainty around the log 2 fold change. P values are from hypothesis test for each ASV to see if there is evidence against the null hypothesis that there is no effect of attractiveness group on the ASV. Benjamini-Hochberg p values are adjusted for false discovery rate.
